# Antimicrobial Stewardship in General Practice: A Scoping Review of the Component Parts

**DOI:** 10.3390/antibiotics9080498

**Published:** 2020-08-09

**Authors:** Lesley Hawes, Kirsty Buising, Danielle Mazza

**Affiliations:** 1 Department of General Practice, School of Primary and Allied Health Care, Monash University, Level 1, 270 Ferntree Gully Road, Notting Hill, Victoria 3168, Australia; Danielle.Mazza@monash.edu; 2 National Centre for Antimicrobial Stewardship, The Peter Doherty Institute for Infection and Immunity, Level 5, 792 Elizabeth Street Melbourne, Victoria 3000, Australia; Kirsty.Buising@mh.org.au; 3 Acting Director, Victorian Infectious Diseases Service, Royal Melbourne Hospital, 300 Grattan St, Parkville, Victoria 3050, Australia

**Keywords:** general practice, ambulatory care, general practitioner, family physician, antimicrobial stewardship, antibiotics, antibiotic prescriptions, health policy, framework

## Abstract

There is no published health-system-wide framework to guide antimicrobial stewardship (AMS) in general practice. The aim of this scoping review was to identify the component parts necessary to inform a framework to guide AMS in general practice. Six databases and nine websites were searched. The sixteen papers included were those that reported on AMS in general practice in a country where antibiotics were available by prescription from a registered provider. Six multidimensional components were identified: 1. Governance, including a national action plan with accountability, prescriber accreditation, and practice level policies. 2. Education of general practitioners (GPs) and the public about AMS and antimicrobial resistance (AMR). 3. Consultation support, including decision support with patient information resources and prescribing guidelines. 4. Pharmacist and nurse involvement. 5. Monitoring of antibiotic prescribing and AMR with feedback to GPs. 6. Research into gaps in AMS and AMR evidence with translation into practice. This framework for AMS in general practice identifies health-system-wide components to support GPs to improve the quality of antibiotic prescribing. It may assist in the development and evaluation of AMS interventions in general practice. It also provides a guide to components for inclusion in reports on AMS interventions.

## 1. Introduction

Antimicrobial stewardship (AMS) may be defined as “a coherent set of actions which promote using antimicrobials responsibly” [1]. An AMS program is “an organisational or healthcare-system-wide approach to promoting and monitoring judicious use of antimicrobials to preserve their future effectiveness” [2]. The primary aim for AMS programs is to improve the safety and quality of patient care. It is important to optimize treatment while minimizing potential harms related to antimicrobial use for both the individual and the population. AMS typically applies to all antimicrobial agents, but this paper will also refer to antibiotics, as they are the most commonly prescribed antimicrobial agents in general practice (family medicine, ambulatory care).

AMS programs are now common in hospitals, but most of the antibiotics consumed by the population are from prescriptions written by general practitioners (GPs) in general practice (also known as ambulatory care) [3,4,5,6], where AMS remains embryonic. Studies of antibiotic use in the community strongly suggest high rates of inappropriate prescribing, particularly unnecessary use for self-limiting illnesses [7,8,9,10]. It is estimated that the escalation of antimicrobial resistance (AMR) will lead to 10 million deaths a year by 2030 [11], thus the need for action on AMS in the community is urgent. However, we do not fully understand what external or local practice factors may be important, nor do we understand the contributions from policy makers and non-prescribing practice team members. The reasons for antibiotic prescriptions are multi-factorial and may include the patient expectation that antibiotics will help manage a viral or self-limiting infection, a lack of alternative treatments, and a mismatch between pack size and prescribing guidelines and GP prescribing and communication habits [12]. Patient populations and health systems differ, thus a variety of approaches at different levels may be required. AMS interventions have been heterogenous, demonstrating little superiority of any intervention or combination of interventions [13,14,15,16]. There is little evidence for the sustainability, acceptability or scalability of interventions [15,16,17,18,19]. Regulatory and cultural environments are not well described [20]. The reasons why interventions do not work are under researched [21].

Identifying the component parts of a framework for AMS in general practice, along with a description of the roles and responsibilities of key stakeholders, is an essential step towards developing an AMS model that can be effectively implemented. Such a framework can also highlight gaps and priorities for AMS in general practice. A preliminary literature search did not find any published existing health system frameworks specific to AMS in general practice. A scoping literature review was therefore chosen, as this can describe the quantity of research in an area, identify gaps that can be addressed through ongoing research and map the key concepts that underpin a research area [22,23,24].

### 1.1. Aim of This Scoping Review

The aim of this scoping review was to identify the health-system-wide component parts of AMS in general practice to inform a framework which may be used to guide activity in this health care context.

### 1.2. Scoping Review Question

What are the core components of general practice AMS frameworks or model frameworks that have been described in the existing published literature?

A secondary question was asked: Which stakeholders have responsibility for governance of general practice AMS?

## 2. Results

**Figure 1:**
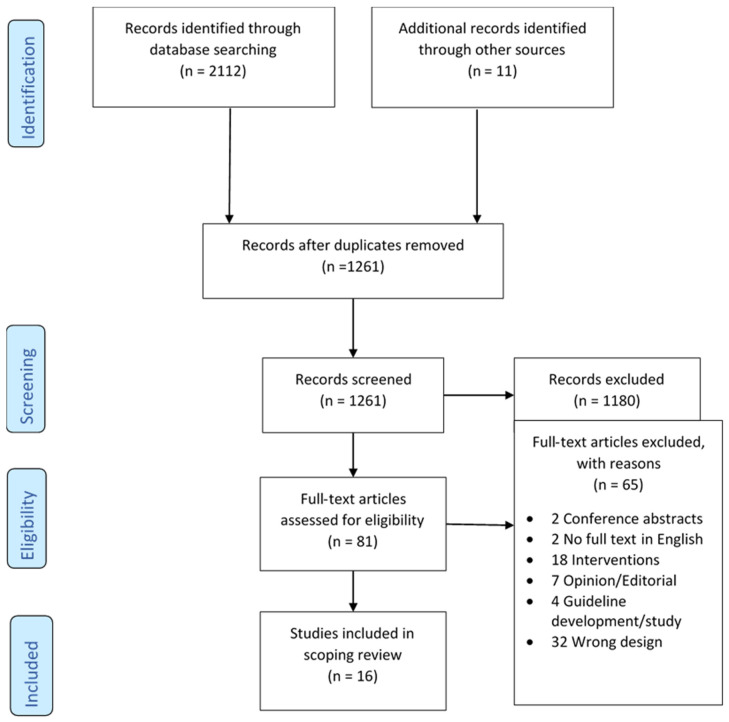
Preferred Reporting Items for Systematic reviews and Meta-Analyses extension for Scoping Reviews (PRISMA-ScR) flow diagram.

The database searches returned 1261 non-duplicate citations, and after title and abstract screening, 81 papers were screened by full text. Five papers were selected from the database searches [25,26,27,28,29]; four papers were identified by searching the references of included papers [30,31,32,33]; one paper from a personal library [20]; and six papers from the website searches [2,34,35,36,37,38]; resulting in 16 papers that were included in the final review (Figure 1).

Seven papers described single-country AMS frameworks: two were from England [25,30], two from Sweden [31,32], two from Australia [26,34] and one from the United States of America (USA) [33]. One paper was a description of general practice AMS in France, which was presented along with an ‘inventory of AMS programs’ from 15 other European countries, the USA, and Canada [29]. One paper detailed the European Union (EU) guidelines for AMS [36] (the EU in 2018 had 28 member states). Two were within the United Kingdom [2,37], the other five papers were not geographically limited [20,27,28,35,38] (Supplementary File 1).

Ten papers described a health-system-wide approach to AMS which included general practice along with other health sectors such as hospital and aged care services [2,25,30,31,32,34,35,36,37,38]. When a component was not clearly identified as applicable to only one part of the health system (e.g., hospital or aged care only) it was assumed that the component was applicable to general practice. The other six papers focused on general practice-specific AMS frameworks [20,26,27,28,29,33], of which two were further limited to the management of respiratory tract infections (RTI) [26,27]. These were included as RTIs account for a large proportion of antibiotic prescribing in general practice [39,40], and one was “envisaged as a prototype that can be adapted to other infections in the long term” [27] (Supplementary File 1).

One paper was published in 2001 [28]; the other 15 papers were published between 2012 and 2018 [2,20,25,26,27,29,30,31,32,33,34,35,36,37,38] (Supplementary File 1).

Funding sources were not stated in nine papers [25,28,29,31,32,34,36,37,38], five papers received non-commercial support [2,20,26,30,33], and two papers received funding from commercial entities [27,35] (Supplementary File 1).

### 2.1. The Identified Components

Using the focus of the scoping review question “What are the core components of general practice AMS frameworks or model frameworks?”, the components were categorized under six broad headings of: governance, education, consultation support, pharmacy and nurse based approaches, monitoring, and research (Table 1). Each has subcomponents. The secondary question of “which stakeholders have responsibility for governance of general practice AMS?” is addressed under governance.

#### 2.1.1. Governance

Governance, including descriptions of strategies, policies, action plans, regulations and responsibility to support AMS in general practice, was reported in several frameworks (Table 1, with examples in Supplementary File 2).

The need for a national action plan or strategy or policies for antimicrobial resistance (AMR) was described in nine papers [27,29,30,31,32,34,35,36,38]. Descriptions of responsibility were often generic, such as “Overall accountability for antimicrobial management lies at the highest level of each health service organisation, and with the clinicians responsible for delivering services efficiently and effectively” [34]. The clearest description of specific responsibility was in Sweden’s AMR program (Strama) “Strama is composed of a national steering group and regional Strama groups in every Swedish county…” [31]. England and Australia have called for commissioning groups [37] or primary care networks to assist [34], and England’s Antimicrobial Stewardship subgroup of the Advisory Committee on Antimicrobial Resistance and Healthcare Associated Infection has a remit which includes the development of AMS tools [30], which may indicate emerging structures.

The World Health Organization (WHO) suggested the inclusion of AMR on the national risk register as “an effective mechanism for cross government commitment” [38]. Ten papers described regulations around antibiotic prescribing as being critical components of community-based AMS activity [2,25,26,27,29,30,34,35,36,38]. Two papers specifically described the accreditation or appraisal of health professionals’ competency to prescribe antibiotics [35,38]. Explicit policies and plans to govern the use of new antibiotics when released were described in four papers, with a focus on curtailing misuse and restricting use to indications of need [2,35,37,38]. The need for practice-level AMS policies was discussed in six papers, although specific examples were limited [2,30,33,34,35,36]. Five papers reported on the need for funding to support AMS activities in general practice, but few details were described about who was responsible for providing this funding or what specifically was funded [32,34,35,36,38].

#### 2.1.2. Monitoring and Feedback

Monitoring (audit, surveillance or tracking), including monitoring of antibiotic prescribing and local patterns of AMR amongst pathogens, was universally included in the frameworks. More specifically, monitoring of antibiotic prescriptions was included in all 16 papers [2,20,25,26,27,28,29,30,31,32,33,34,35,36,37,38], while monitoring of AMR in pathogens was described in 13 papers [2,25,26,27,28,31,32,33,34,35,36,37,38]. Feedback to prescribers was described in 12 papers [2,20,25,26,28,29,31,32,33,34,35,36], but specific examples were limited. Various linkages were described to potentially enhance the utility of this monitoring, including links between prescribing data and antimicrobial resistance, as well as prescribing data links to clinical data including patient demographics, patient management and outcomes data, incidence of infections, and comparisons with prescribing guidelines (Table 1, examples in Supplementary File 2).

#### 2.1.3. Education

The educational activities identified in this scoping review included education of the public and/or patients, as well as continuing education and professional development for the prescribers in general practice (Table 1, examples in Supplementary File 2).

Thirteen papers described the need for public education campaigns to raise awareness of AMR and/or unnecessary use of antibiotics as a core component of an AMS framework [20,26,27,28,29,30,31,32,33,34,35,36,38].

Thirteen papers discussed the importance of providing ongoing education to GPs about AMS and AMR [2,20,26,27,28,29,30,32,33,34,35,36,38]. Six papers described the importance of GPs providing education to patients about appropriate use of antibiotics during a consultation [20,27,33,34,36,37] and nine papers discussed GPs teaching patients to manage self-limiting infections without antibiotics [2,26,27,28,33,34,35,36,37]. Nine papers discussed training GPs to enhance their communication skills; this included training to use patient-centred approaches and shared decision making [2,20,26,27,30,33,34,35,36]. Ten papers described training GPs to use strategies such as delayed prescribing (providing prescriptions to commence only if symptoms worsen and informing patients on how to recognize this) and/or watchful waiting (informing patients about symptoms of concern that should prompt a rapid return for review) [2,20,26,27,29,33,34,35,36,37].

Six papers described education about AMS and AMR for other general practice team members, including practice nurses and community pharmacists [20,28,32,33,34,35].

The promotion and marketing of antibiotics by pharmaceutical companies was recognized as a driver for antibiotic prescribing, and the need for independent education was addressed in six papers [27,28,34,35,36,38].

#### 2.1.4. Consultation Support

Several frameworks discussed providing access to tools and resources that a GP might utilize at the point of care to help inform prescribing decisions. These included: prescribing guidelines; point of care tests and/or laboratory-based investigations including microbiology tests; allergy testing; electronic decision support for prescribers; access to expert advice (such as a clinical microbiologist or infectious diseases specialist phone advice); resources to support shared decision making with patients (Table 1, examples in Supplementary File 2).

The promotion and use of antibiotic prescribing guidelines was described in 12 papers [2,20,27,28,29,31,32,33,34,35,36,38].

Point of care or rapid diagnostic (office-based) tests (e.g., C-reactive protein; influenza antigens, group A streptococcal antigen) were discussed in 11 papers [2,20,26,28,29,31,32,34,35,36,38]. The discussion included both advantages and possible disadvantages to their use [35].

Nine papers addressed the importance of access to suitable microbiology testing and reporting [2,27,28,29,32,34,35,36,38]. This included having access to reliable tests when needed, taking samples correctly, and appropriate review of results. The role of laboratory reporting in guiding the use of antibiotics was also acknowledged, e.g., selective reporting of antimicrobial susceptibilities to direct users to narrow spectrum agents in line with treatment guidelines.

Two papers mentioned access to beta-lactam allergy testing which may help clarify suitable treatment options for the future [34,36].

Electronic decision support for GPs, namely organised patient health and prescribing information to aid decisions, was mentioned in eight papers [2,20,28,29,33,34,35,36].

Access to expert advice was described in six papers [27,29,33,34,35,36]. Two different types of expert advice were mentioned. The first was individual patient specific management advice in which GPs could discuss clinical concerns directly with an expert (e.g., a clinical microbiologist, pharmacist) [33,34,35,36], the second was expert advice for the practice-level AMS program [27,35]. This involved discussion of general strategies for patient management rather than being individual-patient-focused.

Decision support tools for use with patients, including shared decision-making tools (e.g., infographics to guide discussions about options—which might include the natural history of the infection, the likely value of antibiotics, and potential side effects of medications) and patient-focused information about infections and antibiotics (e.g., printed materials), were described in ten papers [2,20,26,28,29,32,33,34,35,36]. Two of the ten papers also mentioned the importance of patient-focused information being available in other languages [29,32].

#### 2.1.5. Pharmacy and Nursing Approaches

These were mainly pharmacy-based, with some recognition of a role for practice-based nurses (Table 1, examples in Supplementary File 2). Pharmacy supply of, and access to, antibiotics was addressed in five papers [30,32,34,36,38]. Pharmacy interventions such as unit-dispensing of medication (dispensing only the prescribed quantity) were mentioned in four papers [2,26,29,36]. Pharmacy review of prescriptions and advice to consumers and health professionals was described in six papers [27,29,33,34,35,36]. Two papers described the disposal of left-over antibiotics as being important [34,36].

A role for practice- or community-based nurses in AMS was described in six papers [20,32,33,34,35,36] (Table 1, with examples in Supplementary File 2). Three papers suggested that nurses could perform a pre-visit triage [20,32,33], two of which were nurse phone call hot lines [32,33], while the third paper described the use of a nurse for pre-visit patient assessment, triage and patient education [20].

#### 2.1.6. Research

The need for targeted, prioritized research into AMR and AMS in the community was addressed in ten papers [2,20,26,28,32,33,34,35,36,38], with specific needs mentioned for implementation research and evaluation of the translation of evidence to practice. Research that recognises the context and culture of general practice and the use of behaviour change science was described in seven papers [2,20,25,27,34,35,36]. Two of these stated that there is no ‘one size fits all’ approach to AMS programs [34,35], and a third noted that “Few studies focused on the organization component of the work system model or the structures and roles that organize a clinic” [20] (Table 1, examples in Supplementary File).

## 3. Discussion

Our scoping review of the literature on frameworks for AMS in general practice found the core components to be: 1. Governance. 2. Monitoring of antibiotic prescribing and AMR with feedback to GPs. 3. Education of the public and health professionals about AMR and AMS. 4. Consultation support. 5. Pharmacy- and nursing-based approaches. 6. Research.

The lack of clear descriptions about who was responsible for implementing and coordinating these activities was striking. National-level responsibility for the monitoring of antibiotic resistance and prescribing was described, but there was no clear description of any governing body responsible for all aspects of this framework, with the exception perhaps of Strama in Sweden [31,32]. England and Australia have called for commissioning groups or primary care networks to assist [34,37], and England’s Antimicrobial Stewardship subgroup of the Advisory Committee on Antimicrobial Resistance and Healthcare Associated Infection has a remit which includes the development of AMS tools [30], which may indicate emerging structures. There was no clear description of GPs’ perceptions about governance or clinical autonomy.

Monitoring of antimicrobial resistance and prescribing was almost universal but, apart from Sweden’s Strama program [31,32], it was not clear who should provide the analysis and regular feedback to GPs, where the data should be published or what GPs’ perceptions were of the monitoring process or feedback. Where GPs were to analyse their own prescribing, it was not stated how patients should be selected, which leaves open the possibility of selection bias, and there were few descriptions of what GPs should be monitoring.

While there were calls for health professional education on AMS, apart from noting that pharmaceutical companies should not be responsible for this, no mandatory education programs were described, nor was it clear who should be responsible for the development, delivery and evaluation of education programs, or to what extent AMS education should be provided to general practice support staff. Similarly, GPs were called upon to educate patients about management and treatment of their infections (including non-antibiotic management and treatment), with patient information leaflets and posters of the main aids offered. It was not clear who should develop these, what should be included, or how to check that they met basic literacy standards or that different language versions were checked for cultural appropriateness. Public awareness campaigns about AMR and AMS occurred but were not well described. It was recognised that expert advice regarding general practice antibiotic prescribing decisions may be useful but is difficult to arrange during a consultation. The only description of an established expert advice program for general practitioners was telephone advice in France [29].

Pharmacist- and nurse-based approaches were poorly described. Their roles in AMS and that of the general practice team needs further research. To ensure consistent messages are provided to patients, AMS programs may benefit by including all general practice staff and community providers.

In research, customising interventions for the context and culture of the health service were recognised as critical to the success of AMS programs. Factors such as practice size and time for appointments [41], patient age, GP–patient relationship, being located in a rural area and socio-economic status affect antibiotic prescribing rates [42,43,44]. Local barriers and enablers may partly explain variation in AMS outcomes. For example, a GP with a high workload and few resources may find it easier to prescribe a requested antibiotic than to attempt to educate the patient about why they do not need an antibiotic for that condition. Social science and behaviour change principles would also appear to be important in the development of future AMS interventions [2,20,25,27,34,35,36].

There are several potential limitations to this review. The search strategy may have missed studies which were not indexed under the search terms. Only a limited search was made for grey literature and all papers were restricted to the English language, with eight papers (50%) from Europe. The selected papers may not have included a full description of their AMS frameworks—one paper explicitly excluded public awareness of AMR and disposal of waste medicines [2], but others may not have stated their exclusions. One reviewer conducted the screening and extraction, which may have introduced selection bias. All three authors provided input into the development of the framework. Scoping reviews do not rate the quality of the evidence [45], and the included papers reported few challenges with implementing frameworks, such as resistance from GPs. Thus, implications for policy cannot be graded [45]. This scoping review may have limited applicability for other primary care community prescribers, e.g., dentists, nurse practitioners or pharmacists, and in countries that were not represented in the papers assessed, including countries where antibiotics are available without prescription. Identification of resources to support the identified components, such as educational resources, was beyond the scope of this research.

Interestingly, this review demonstrated that none of the selected papers had articulated the framework in this way. This may be because the evidence for AMS in general practice is still emerging. Although the core elements of the framework appear to have face validity, the method did not enable the authors to examine possible inter-dependencies between components, or examine whether components should be introduced in any order. Missing components or unexplored interdependencies may partly explain why AMS interventions have succeed in some contexts but not in others [17,46]. Possible synergy between the diverse components [26] may explain why multi-faceted interventions were more likely to be successful in reducing antibiotic prescribing [17,47], e.g., it is possible that GPs and/or communities require access to a range of resources. Further research amongst relevant stakeholders is required to determine the validity of these components and to determine the framework’s utility for the development, evaluation and reporting of AMS interventions in general practice.

## 4. Materials and Methods

The scoping review was conducted according to the Joanna Briggs Institute’s standardised method [45]. Selection criteria were developed a priori then iteratively refined to capture papers that answered the scoping review question.

### 4.1. Selection Criteria

To be included, the paper had to describe an AMS framework that was applicable to GPs working in a community-based general practice, in a country with a developed health care system where systemic antibiotics are primarily available by prescription from a registered provider (e.g., OECD country). All eligible publications were included even if there were multiple publications about the same framework, but with varied analysis (e.g., improvements to or sustainability of the framework). Publications which included, e.g., hospitals and aged care were included if they described a health-system-wide approach to AMS which included general practice.

The search strategy excluded AMS activities that targeted only:

- Hospitals, including their emergency departments and outpatient (specialist) clinics, residential care including nursing or aged care homes; veterinary clinics;
- Other community prescribers (e.g., nurse practitioners, dentists, other medical specialists, veterinarians);
- Patients or community members; animals; the environment;
- Settings where antibiotics were frequently available without a prescription.

Reports about antibiotic usage or AMR; clinical guidelines on infections and their treatment; the development, use of and/or promotion of antibiotic prescribing guidelines; the development of new antibiotics or vaccines; infection prevention, the relationship between antibiotic use and resistance; the economic burden of resistance were also excluded. Reports about interventions were excluded if they did not also describe the health system context in which they were carried out.

The search was limited to English language documents, and no time limits were imposed.

### 4.2. Search Strategy

The Ovid Medline database was searched to identify relevant keywords and index terms. The identified keywords and index terms were then used to search the Embase, Ovid Medline, Scopus, CINHAL, PsychINFO and Cochrane databases from inception to September 2018. Pre-determined search terms included the headings (with synonyms) for antibiotics AND antibiotic prescriptions AND general practitioners AND general practice AND stewardship AND framework (the search strategy is provided in Supplementary File 3). The reference lists of included studies and personal libraries were also reviewed.

A limited English-language grey literature search examined the websites of the Australian Commission on Safety and Quality in Health Care, Royal Australian College of General Practitioners, the European Centre for Disease Prevention and Control (ECDC), England’s National Health Service, the National Institute for Health and Care Excellence, the Royal College of General Practitioners, the British Society for Antimicrobial Chemotherapy, USA’s Centers for Disease Control and Prevention (CDC), and the World Health Organization (Supplementary File 3); searching for ‘antibiotic’ or ‘antimicrobial stewardship’, or ‘general practice’ or ‘family medicine’ and included papers if they met the inclusion and exclusion criteria.

### 4.3. Data Collection, Charting and Identification of AMS Components

Database citations were downloaded to Covidence [48] and duplicates removed. Titles and abstracts were reviewed for inclusion, followed by full text screening. This was done by one author (LH) with a second reviewer (DM) available for discussion where required. The full texts of the selected references were uploaded into NVivo 12 Plus [49] for coding by one author (LH). Each text was read through, then analysed thematically using line-by-line inductive coding [50]. All three authors then developed and refined the coding into themes. This involved inductive analysis using repetition of themes across the papers [50,51] and deductive/a priori analysis based on experience in hospital AMS programs (KB) and general practice quality improvement programs (DM and LH). Component parts were mapped onto a table developed for this review with input from all three authors.

## 5. Conclusions

This manuscript reviews the existing literature on general practice AMS frameworks and describes, for the first time, a comprehensive multifaceted framework with the potential to focus attention on neglected areas in AMS in general practice. The articulation of the six core components into an actionable framework should help guide future activity to strengthen AMS in general practice. It not only provides a framework to guide AMS activity, it also provides a guide to the components that may be considered and reported in future publications about AMS interventions. Gaps in the AMS framework are highlighted, including that identification of responsibility for the components was lacking, as were the perceptions of GPs.

## Figures and Tables

**Table 1:** AMS in general practice: Chart of identified component parts.

Component/Author, Date	Ashiru-Oredope, 2012 [30]	Ashiru-Oredope, 2013 [25]	ACSQHC, 2018 [34]	BSAC, 2018 [35]	Del Mar, 2017 [26]	Essack, 2013 [27]	European Commission, 2017 [36]	Keller, 2018 [20]	McNulty, 2001 [28]	Molstad, 2008 [31]	Molstad, 2017 [32]	NICE, 2015 [2]	Sanchez, 2016 [33]	UK Faculty [37]	Wang, 2015 [29]	WHO, 2015 [38]
**2.1.1. Governance**																
National action plan, policy or strategy	x		x	x		x	x			x	x				x	x
AMR included on national risk register																x
Regulations around AMS and antibiotic prescribing	x	x	x	x	x	x	x					x			x	x
Accreditation of prescribers				x												x
Funding for AMR/AMS			x	x			x				x					x
Planning for release of new antibiotics				x								x		x		x
Practice level AMS policy/program/activities	x		x	x			x					x	x			
**2.1.2. Monitoring and Feedback**																
Monitoring of antibiotic prescriptions	x	x	x	x	x	x	x	x	x	x	x	x	x	x	x	x
Monitoring of antimicrobial resistance		x	x	x	x	x	x		x	x	x	x	x	x		x
Feedback to prescribers and reporting		x	x	x	x		x	x	x	x	x	x	x		x	
**2.1.3. Education**																
Community and patient education about AMR and AMS	x		x	x	x	x	x	x	x	x	x		x	x	x	x
GP continuing education in AMS and AMR	x		x	x	x	x	x	x	x		x	x	x		x	x
GP education on communication skills, patient-centred approaches and shared decision making	x		x	x	x	x	x	x				x	x			
GP education on non-antibiotic management of self-limiting infection			x	x	x	x	x		x			x	x	x		
GP education on delayed prescribing/watchful waiting			x	x	x	x	x	x				x	x	x	x	
General practice team member education			x	x				x	x		x		x			
Independent education (restrict pharma marketing)			x	x		x	x		x							x
**2.1.4. Consultation Support**																
Prescribing guidelines			x	x		x	x	x	x	x	x	x	x		x	x
Point of care tests			x	x	x		x	x	x	x	x	x			x	x
Microbiology testing and reporting			x	x		x	x		x		x	x			x	x
Allergy testing			x				x									
Electronic decision support for prescribers			x	x			x	x	x			x	x		x	
Expert advice			x	x		x	x						x		x	
Decision support for use with patients			x	x	x		x	x	x		x	x	x		x	
**2.1.5. Pharmacy and Nursing Approaches**																
Unit dispensing					x		x					x			x	
Supply of and timely access to antibiotics	x		x				x				x	NA				x
Pharmacy review and advice			x	x		x	x						x		x	
Appropriate disposal of left-over antibiotics			x				x					NA				
Nurse triage, patient assessment and education			x	x			x	x			x		x			
**2.1.6. Research**																
Research into AMR/AMS gaps, translation into practice			x	x	x		x	x	x		x	x	x			x
Research into context, culture of general practice and behaviour change strategies		x	x	x		x	x	x			x					

Abbreviations: AMR Antimicrobial resistance; AMS antimicrobial stewardship; GP general practitioner; NA: Not applicable/excluded in this paper.

## Supplementary Materials

The following are available online at https://www.mdpi.com/2079-6382/9/8/498/s1, File S1: The characteristics of the included papers. File S2: Examples of the components. File S3: The search strategy.
