# Supplementary material for: Antimicrobial Stewardship in General Practice: A Scoping Review of the Component Parts
Source: Antibiotics (Basel). 2020 Aug 9;9(8):498. doi: 10.3390/antibiotics9080498 (PMC7459857; doi:10.3390/antibiotics9080498)
Supplement: Supplementary file 1 [file antibiotics-09-00498-s001.pdf]

Supplementary file 1 Characteristics of the included papers

First author, year of publication	Country(s) for application	Lead organisations	Aim or purpose of framework	Methodology of paper	Intended audience	Stage of framework	GP input into or support for framework	Funding sources
Ashiru-Oredope, 2012[1]	England	Department of Health's Advisory Committee on Antimicrobial Resistance and Healthcare Associated Infection.	This paper describes the development of new antimicrobial stewardship programmes for primary care and hospitals by the Department of Health's Advisory Committee on Antimicrobial Resistance and Healthcare Associated Infection: Antimicrobial Stewardship in Primary Care Initiative.	Narrative description of government initiative	Not stated	Description of components being implemented	RCGP supported	MIHR, CIPM, CPSSQ/NIHR, BSAC, British Infection Association

First author, year of publication	Country(s) for application	Lead organisations	Aim or purpose of framework	Methodology of paper	Intended audience	Stage of framework	GP input into or support for framework	Funding sources
Ashiru-Oredope, 2013[2]	England	English Surveillance Programme for Antimicrobial Utilization and Resistance (ESPAUR).	Public Health England has developed a new national programme, the English Surveillance Programme for Antimicrobial Utilization and Resistance (ESPAUR). The programme will bring together the elements of antimicrobial utilization and resistance surveillance in both primary and secondary care settings, alongside the development of quality measures and methods to monitor unintended outcomes of antimicrobial stewardship and both public and professional behaviour interventions. This article reports on the background to the programme development, the current oversight group membership and the public reporting structure.	Narrative description of government initiative	Not stated	Description of components being implemented	RCGP represented	Not stated

First author, year of publication	Country(s) for application	Lead organisations	Aim or purpose of framework	Methodology of paper	Intended audience	Stage of framework	GP input into or support for framework	Funding sources
ACSQHC, 2018[3]	Australia	Australian Commission on Safety and Quality in Health Care	This publication is designed to provide clinicians and managers working in all healthcare sectors with the evidence, expert guidance and tools they need to initiate and sustain AMS activities in a diverse range of practice settings – hospitals (public and private, metropolitan and rural), primary care and aged care homes	Chapters 1–7 provide strategies for implementing and sustaining AMS. Chapters 8–12 examine the roles of the different clinicians in AMS.	Clinicians and managers working in all healthcare sectors.	Recommended framework	Advice and review	Not stated
BSAC, 2018[4]	Global workbook for AMS across health system	British Society for Antimicrobial Chemotherapy [BSAC]	E-book on Global Antimicrobial Stewardship that is relevant to health care professions working in preventing and managing infection across the healthcare communities and health care facilities	Interactive e-book on global AMS, building on a massive open on-line stewardship course.	It aims to support health care professionals, or teams, or policy makers interested in learning about bringing the principles of stewardship to the bed side.	Theoretical model with real-world case studies	Not stated	Alere now Abbott, Accelerate Diagnostics, MSD, Pfizer Inc
Del Mar, 2017[5]	Australia	Bond Uni, Uni Qld, TG, NPS	The aim of this narrative review is to describe interventions that, if implemented on a national scale and successfully lowered the volume of antibiotics prescribed in general practice for ARIs, should reduce community-acquired antibiotic resistance.	Narrative review of literature and discussion at national roundtable meeting in 2017.	Australian researchers, policy makers and organisations came together to discuss ways of dealing with the antibiotic resistance crisis from a general practice perspective.	Theoretical model	Lead author. GPs attendees at the national roundtable.	NHMRC for CRE-MAR

First author, year of publication	Country(s) for application	Lead organisations	Aim or purpose of framework	Methodology of paper	Intended audience	Stage of framework	GP input into or support for framework	Funding sources
Essack, 2013[6]	Global	In collaboration with the Global Respiratory Infection Partnership: Attila Altiner (Germany), John Bell (Australia), Martin Duerden (UK), Sabiha Essack (South Africa), Roman Kozlov (Russia), John Oxford (UK), Antonio Carlos Pignatari (Brazil), Aurelio Sessa (Italy), Alike van der Velden (The Netherlands).	The global respiratory infection partnership has formulated a pentagonal (five P) framework for the non-antibiotic management of upper respiratory tract infections (URTIs) – one of the most common conditions in primary care for which antibiotics are prescribed.	Framework developed by GRIP and reviewed by healthcare professionals in 18 countries.	The global framework is strengthened through a collaborative approach by multiple primary healthcare provider specialties and is applicable across countries and continents. It is envisaged as a prototype that can be adapted to other infections in the long term	Theoretical model	Collaboration with primary healthcare provider specialties	Reckitt Benckiser Group PLC Editorial assistance during the development and revision of this manuscript was provided by Mash Health Limited and supported by Reckitt Benckiser Group PLC. The authors provided substantial contributions to the development and revision of the manuscript and approved the final version of the manuscript.

First author, year of publication	Country(s) for application	Lead organisations	Aim or purpose of framework	Methodology of paper	Intended audience	Stage of framework	GP input into or support for framework	Funding sources
European Commission, 2017[7]	Europe	European Centre for Disease Prevention and Control	These guidelines aim to reduce inappropriate use and promote prudent use of antimicrobials. The guidelines are complementary to infection prevention and control guidelines which may exist at national level.<br>These guidelines are intended to be used to inform and assist activities to promote the prudent use of antimicrobials in humans.	These Guidelines on prudent use of antimicrobials in human health are based on a technical report prepared by the European Centre for Disease Prevention and Control (ECDC) with input from EU Member States experts and stakeholders, which should be referred to for details of the methodology used in creating the guidelines as well as for additional references	They target all actors who are responsible for, or play a role in, antimicrobial use and whose contribution is necessary to ensure that antimicrobials are used appropriately.	Recommended model	Not stated	Not stated

First author, year of publication	Country(s) for application	Lead organisations	Aim or purpose of framework	Methodology of paper	Intended audience	Stage of framework	GP input into or support for framework	Funding sources
Keller, 2018[8]	Global Systematic literature review	USA: Johns Hopkins University School of Medicine; Agency for Healthcare Research and Quality; University of Pennsylvania Perelman School of Medicine; North western University Feinberg School of Medicine	Our aim in using this approach was to identify how aspects of the clinic work system could affect AS intervention successes. [Set within "a wider external environment"]	Systematic literature review. 42 quantitative and 17 qualitative articles met the eligibility criteria, with 1 study considered both quantitative and qualitative. We evaluated identified sources and recorded study design and aspects of the studies that could be interpreted in the context of the SEIPS 2.0 model. We also described how measures and outcomes of the studies could be interpreted in the context of the SEIPS 2.0 work system.<br>The SEIPS 2.0 work system includes 5 components within a wider external environment: (1) person(s), (2) tools and technologies, (3) organization, (4) tasks, and (5) physical environment.	Not stated	Theoretical model developed using a human engineering framework to incorporate the entire work system	Not stated	Agency for Healthcare Research and Quality. SCK received funding from the National Center for Advancing Translational Sciences/Johns Hopkins Institute for Clinical and Translational research.

First author, year of publication	Country(s) for application	Lead organisations	Aim or purpose of framework	Methodology of paper	Intended audience	Stage of framework	GP input into or support for framework	Funding sources
McNulty, 2001[9]	Global	UK: Gloucester Royal Hospital	Improving appropriate use of antimicrobials in primary care will require a multi-faceted approach including not only education of primary care health professionals, but also a wider evidence base underlying management of infections, prescribing advice and support, monitoring of antibiotic prescribing and resistance and increasing public awareness.	Narrative literature review	Not stated	Theoretical model	Not stated	Not stated
Molstad, 2008[10]	Sweden	Strama (Swedish Strategic Programme for the Rational Use of Antimicrobial Agents and Surveillance of Resistance)	The aim of this review is to describe the Strama programme and summarise the results of the first 10 years.	Description of Sweden's Strama (AMS) program across primary care, hospital care, nursing homes, and day-care centres.	Not stated	Report of Strama after 10 years of implementation	Represented in regional Strama groups	Not stated
Molstad, 2017[11]	Sweden	Strama	This paper outlines the stepwise development of the strategic programme against antibiotic resistance in Sweden over a period of 20 years.	We describe the structure, key functions and interventions of the initiative across different working areas in human medicine.	The work described here and the lessons learnt could inform countries implementing their own national action plans against antibiotic resistance.	Report of Strama after 20 years of implementation	Represented in local multi-professional groups	Not stated

First author, year of publication	Country(s) for application	Lead organisations	Aim or purpose of framework	Methodology of paper	Intended audience	Stage of framework	GP input into or support for framework	Funding sources
NICE, 2015[12]	England, Wales	National Institute for Health and Care Excellence	The purpose of this guideline is to provide good practice recommendations on systems and processes for the effective use of antimicrobials.	Scoping search, systematic literature review, meta-analysis, call for evidence, discussed by multi-disciplinary group, reviewed clinical and cost-effectiveness, recommendations made. 4-week public consultation.	This guideline may be of interest to adults, young people and children (including neonates) using antimicrobials or those caring for these groups. This includes people and organisations involved with the prescribing and management of antimicrobials in health and social care settings.	Recommended model	Yes	NICE commissioned the NICE Medicines and Prescribing Centre to produce this guideline.

First author, year of publication	Country(s) for application	Lead organisations	Aim or purpose of framework	Methodology of paper	Intended audience	Stage of framework	GP input into or support for framework	Funding sources
Sanchez, 2016[13]	USA	Centers for Disease Control and Prevention	The Core Elements of Outpatient Antibiotic Stewardship provides a framework for antibiotic stewardship for outpatient clinicians and facilities that routinely provide antibiotic treatment.	CDC's Core Elements of Outpatient Antibiotic Stewardship were developed through a combination of consolidating evidence-based antibiotic stewardship practices and building on or adapting known best practices for antibiotic stewardship across other clinical settings, such as the core elements outlined for hospitals and nursing homes. A narrative review of evidence on outpatient antibiotic stewardship interventions, policies, and practices through May 2016 was conducted…Subject-matter experts were asked for specific feedback on the feasibility, acceptability, recommended supplementary materials, and potential for the core elements to promote effective and meaningful improvements in outpatient antibiotic prescribing.	The intended audiences for this guidance include clinicians (e.g., physicians, dentists, nurse practitioners, and physician assistants) and clinic leaders in primary care, medical and surgical specialties, emergency departments, retail health and urgent care settings, and dentistry, as well as community pharmacists, other health care professionals, hospital clinics, outpatient facilities, and health care systems involved in outpatient care	Recommended model	Subject matter experts were identified with expertise in pediatrics, internal medicine, family medicine, emergency medicine, infectious diseases, and pharmacy.	CDC did not accept commercial support for this continuing education activity.

First author, year of publication	Country(s) for application	Lead organisations	Aim or purpose of framework	Methodology of paper	Intended audience	Stage of framework	GP input into or support for framework	Funding sources
UK Faculty of Public Health.[14]	UK	UK Faculty of Public Health, Royal College of Physicians, Royal Pharmaceutical Society, Royal College of Nursing, Royal College of General Practitioners.	Our recommendations for action focus on those areas where there is the potential for immediate action.	Not described	The professional bodies supporting this joint statement consider that action must be taken collegiately by the professions, commissioners, service providers, quality assurance bodies and regulators across the UK to reduce the threat of AMR. Leadership and action must be taken at local, regional, national and international level in support of the AMR strategy, and to tackle this issue in a concerted manner.	Recommended model	Yes	Not stated

First author, year of publication	Country(s) for application	Lead organisations	Aim or purpose of framework	Methodology of paper	Intended audience	Stage of framework	GP input into or support for framework	Funding sources
Wang, 2015[15]	France and 17 countries of Europe and North America.	France: CHU de Nancy–Hôpitaux de Brabois; université de Lorraine.	We had for aim to identify measures implemented in France and abroad for antibiotic stewardship in general practice.	A literature review was conducted from January 2000 to July 2014. Emails were sent to every infectious diseases department, to all regional health authorities (ARS), to the health insurance offices (CPAM) with the highest and lowest antibiotic use, and to the ministry of health to make an inventory of all antibiotic stewardship programs. The ministry of health, the board of general practitioners, infectious diseases specialists, pharmacists, and the medical and pharmacy schools of the nation's capital were contacted in 17 countries of Europe and North America.	Our results could be useful to guide policy for antibiotic stewardship in France.	Case study model	Not stated	Not stated

First author, year of publication	Country(s) for application	Lead organisations	Aim or purpose of framework	Methodology of paper	Intended audience	Stage of framework	GP input into or support for framework	Funding sources
WHO, 2015[16]	Global	World Health Organization	This global action plan provides the framework for national action plans to combat antimicrobial resistance. The overall goal of the action plan is to ensure, for as long as possible, continuity of the ability to treat and prevent infectious diseases with effective and safe medicines that are quality-assured, used in a responsible way, and accessible to all who need them.	The Secretariat used the recommendations of the Strategic and Technical Advisory Group on antimicrobial resistance, existing national and regional action plans, WHO's guidance and action plans on related subjects, as well as other available evidence and analysis… the Strategic and Technical Advisory Group considered input from more than 30 additional participants, including representatives of intergovernmental organizations, civil society, public health and regulatory agencies, industry associations, professional organizations and patient groups…Member States, stakeholders and the Secretariat convened additional high-level technical, political and interagency discussions to contribute to the action plan.	The framework presented below tabulates the actions that the Member States, Secretariat and international and national partners need to take in order to attain the goal and meet the objectives of the global plan.	Recommended model. It is expected that countries will develop their own national action plans on antimicrobial resistance in line with the global plan.	Not stated	Not stated

References

1. Ashiru-Oredope, D.; Sharland, M.; Charani, E.; McNulty, C.; Cooke, J.; ARHAI Antimicrobial Stewardship Group. Improving the quality of antibiotic prescribing in the NHS by developing a new Antimicrobial Stewardship Programme: Start Smart - Then Focus. *J. Antimicrob. Chemother.* **2012**, *67 Suppl 1*, i51-63, doi:10.1093/jac/dks202.

2. Ashiru-Oredope, D.; Hopkins, S.; English Surveillance Programme for Antimicrobial Utilization Resistance Oversight Group. Antimicrobial stewardship: English Surveillance Programme for Antimicrobial Utilization and Resistance (ESPAUR). *J. Antimicrob. Chemother.* **2013**, *68*, 2421-2423, doi:10.1093/jac/dkt363.
3. Australian Commission on Safety and Quality in Health Care. Antimicrobial stewardship in Australian health care. ACSQHC: Sydney, 2018.
4. British Society for Antimicrobial Chemotherapy; ESCMID Study Group for Antimicrobial Stewardship; European Society of Clinical Microbiology and Infectious Diseases. Antimicrobial stewardship: from principles to practice. BSAC: Birmingham, 2018.
5. Del Mar, C.B.; Scott, A.M.; Glasziou, P.P.; Hoffmann, T.; van Driel, M.L.; Beller, E.; Phillips, S.M.; Dartnell, J. Reducing antibiotic prescribing in Australian general practice: time for a national strategy. *Med. J. Aust.* **2017**, *207*, 401-406, doi:10.5694/mja17.00574.
6. Essack, S.; Pignatari, A.C. A framework for the non-antibiotic management of upper respiratory tract infections: towards a global change in antibiotic resistance. *Int. J. Clin. Pract. Suppl.* **2013**, *67* 4-9, doi:10.1111/ijcp.12335.
7. European Commission. EU Guidelines for the prudent use of antimicrobials in human health. ECDC: 2017.
8. Keller, S.C.; Tamma, P.D.; Cosgrove, S.E.; Miller, M.A.; Sateia, H.; Szymczak, J.; Gurses, A.P.; Linder, J.A. Ambulatory antibiotic stewardship through a human factors engineering approach: a systematic review. *J. Am. Board Fam. Med.* **2018**, *31*, 417-430, doi:10.3122/jabfm.2018.03.170225.
9. McNulty, C.A. Optimising antibiotic prescribing in primary care. *Int. J. Antimicrob. Agents* **2001**, *18*, 329-333, doi:10.1016/s0924-8579(01)00412-5.
10. Molstad, S.; Erntell, M.; Hanberger, H.; Melander, E.; Norman, C.; Skoog, G.; Lundborg, C.S.; Söderström, A.; Torell, E.; Cars, O. Sustained reduction of antibiotic use and low bacterial resistance: 10-year follow-up of the Swedish Strama programme. *Lancet Infect. Dis.* **2008**, *8*, 125-132, doi:10.1016/s1473-3099(08)70017-3.
11. Molstad, S.; Lofmark, S.; Carlin, K.; Erntell, M.; Aspevall, O.; Blad, L.; Hanberger, H.; Hedin, K.; Hellman, J.; Norman, C., et al. Lessons learnt during 20 years of the Swedish strategic programme against antibiotic resistance. *Bull. World Health Organ.* **2017**, *95*, 764-773, doi:10.2471/BLT.16.184374.
12. National Institute for Health and Care Excellence. Antimicrobial stewardship: systems and processes for effective antimicrobial medicine use. Full guideline: methods, evidence and recommendations. In *NICE guideline*, NICE: 2015.
13. Sanchez, G.V.; Fleming-Dutra, K.E.; Roberts, R.M.; Hicks, L.A. Core elements of outpatient antibiotic stewardship. *MMWR Recomm. Rep.* **2016**, *65*, 1-12, doi:10.15585/mmwr.rr6506a1.
14. The UK Faculty of Public Health; The Royal College of Physicians; The Royal Pharmaceutical Society; The Royal College of Nursing; The Royal College of General Practitioners. Joint statement on antimicrobial resistance. FPH, RCP, RPS, RCN, RCGP: 2014.
15. Wang, S.; Pulcini, C.; Rabaud, C.; Boivin, J.M.; Birge, J. Inventory of antibiotic stewardship programs in general practice in France and abroad. *Med. Mal. Infect.* **2015**, *45*, 111-123, doi:10.1016/j.medmal.2015.01.011.
16. World Health Organization. Global action plan on antimicrobial resistance. WHO: Geneva, 2015.

Supplementary file 2 Examples of components and sub-components

Component	Examples
**2.1.1. Governance**	
**National action plan, policy or strategy; identification of responsibility**	[In 1999 the Department of Health] Set out an action plan for the NHS, aimed at reducing the emergence and spread of antimicrobial resistance and its impact on the treatment of infection. Includes strategies to monitor and optimize antimicrobial prescribing by implementing antibiotic guidelines, supporting professional development on appropriate prescribing, reducing inappropriate prescribing and using clinical governance arrangements to support improved prescribing… [1].<br>Policy measures to advance appropriate, rational antibiotic use need to be country-specific and tailored to local circumstances including, but not limited to, the prevailing burden of disease, taking into account underlying comorbidities, such as HIV and AIDS, and existing resistance rates [2].<br>At the national level, operational action plans to combat antimicrobial resistance are needed to support strategic frameworks. All Member States are urged to have in place, within two years of the endorsement of the action plan by the Health Assembly, national action plans on antimicrobial resistance that are aligned with the global action plan and with standards and guidelines established by intergovernmental bodies... These national action plans are needed to provide the basis for an assessment of the resource needs, and should take into account national and regional priorities [3].<br><br>**Secondary question: Which stakeholders have responsibility for governance of general practice AMS?**<br>Establish clear governance arrangements. The Australian Government Department of Health and Department of Agriculture and Water Resources are responsible for the National Antimicrobial Resistance Strategy…Overall accountability for antimicrobial management lies at the highest level of each health service organisation, and with the clinicians responsible for delivering services efficiently and effectively… [4].<br>Policymakers are called upon to create an environment where the use of antibiotics is not the norm, by introducing disincentives to antibiotic use and surveillance programs, along with guidance that encourages and promotes self-management with symptomatic medications as the treatment in the first instance [2].<br>Strama is composed of a national steering group and regional Strama groups in every Swedish county [5].<br>Implementation of this guidance is the responsibility of local commissioners and/or providers. Commissioners and providers are reminded that it is their responsibility to implement the guidance, in their local context…Ensure that roles, responsibilities and accountabilities are clearly defined within an antimicrobial stewardship programme [6].<br>The professional bodies supporting this joint statement consider that action must be taken collegiately by the professions, commissioners, service providers, quality assurance bodies and regulators across the UK to reduce the threat of AMR. Leadership and action must be taken at local, regional, national and international level in support of the AMR strategy, and to tackle this issue in a concerted manner [7].
**AMR included on national risk register**	Recognize antimicrobial resistance as a priority need for action across all government ministries through inclusion in national risk registers or other effective mechanisms for cross government commitment [3].
**Regulations around AMS & antibiotic prescribing**	National, regional and local governments… responsibilities include legislation, regulation and auditing compliance with legal, policy and professional standards. [8].<br>Provide leadership to strengthen medicines regulatory systems at national and regional levels, so that appropriate practices for optimizing use of antimicrobial medicines are supported by appropriate and enforceable regulation, and that promotional practices can be adequately regulated [3].
**Accreditation of prescribers**	In the United Kingdom (UK) it is recommended that defined antimicrobial prescribing and stewardship competencies are incorporated into appraisals for prescribers. [9].<br>Professional organizations and societies should establish antimicrobial resistance as a core component of education, training, examination, professional registration or certification, and professional development…distribution, prescription, and dispensing of antimicrobials is carried out by accredited health or veterinary professionals under statutory body supervision…[3].
**Funding for AMR/AMS activities**	Sustainable funding is required to allocate time for clinical experts to work closely with prescribers. A mandate and financial support from the government is needed [10].<br>Member States should consider assessing investment needs for implementation of their national action plans on antimicrobial resistance, and should develop plans to secure and apply the required financing [3].

**Planning for release of new antibiotics**	Consider using multiple approaches to support the introduction of a new antimicrobial, including: electronic alerts to notify prescribers about the antimicrobial; prescribing guidance about when and where to use the antimicrobial in practice; issuing new or updated formulary guidelines and antimicrobial prescribing guidelines; peer advocacy and advice from other prescribers; providing education or informal teaching on ward rounds; shared risk management strategies for antimicrobials that are potentially useful but may be associated with patient safety incidents [6].
**Practice level AMS policy and program**	The stewardship programme is established with clear lines of accountability and there is a structure within the organisation/setting that can allow the implementation of a stewardship programme to take place, support the scheme, monitor its performance and hold it to account for performance and outcome measures… [9].<br>Outpatient clinicians and clinic leaders can implement policies and interventions to promote appropriate antibiotic prescribing practices. A stepwise approach with achievable goals can facilitate policy and practice changes and help clinicians and staff members from feeling overwhelmed. [11].
**2.1.2. Monitoring and feedback**	
**Monitoring of antibiotic prescriptions**	Understanding local, regional and national variation in antimicrobial prescribing is essential for assessing the impact of interventions to change prescribing behaviour. Prescribing data need to be linked to antimicrobial resistance data and patient outcomes to ensure that both positive and negative potential outcomes are evaluated [12].<br>Consider using the following antimicrobial stewardship interventions: review of prescribing by antimicrobial stewardship teams to explore the reasons for increasing, very high or very low volumes of antimicrobial prescribing, or use of antimicrobials not recommended in local (where available) or national guidelines…Consider developing local systems and processes for peer review of prescribing. Encourage an open and transparent culture that allows health professionals to question antimicrobial prescribing practices of colleagues when these are not in line with local (where available) or national guidelines and no reason is documented [6].<br>When setting up tracking and reporting systems, decisions need to be made about the level at which to track and report (i.e., at the individual clinician level or at the facility level), which outcomes to track and report, and how to obtain the data for tracking and reporting. … Analysis can occur at the individual clinician level or at the facility level (i.e., aggregate of all clinician antibiotic prescriptions). The preferred approach, when possible, is to track antibiotic prescribing at the individual clinician level…Systems also can track the percentage of visits for which an individual clinician prescribes antibiotics (e.g., number of all antibiotics prescribed for all diagnoses by a clinician divided by the total number of visits for all diagnoses for that clinician). [11].<br><br>**Secondary question: Which stakeholders have responsibility for monitoring of antibiotic prescribing in the general practice setting?**<br>Strama groups in every Swedish county (panel). The national Strama group includes a broad representation of professional organisations and relevant authorities. The main objectives of the national group are to coordinate activities for the containment of antibiotic resistance at the national level. Activities include the analysis of trends in antibiotic resistance and consumption [5].<br>MedQual …is a network dedicated to monitoring antibiotic use and antibiotic resistance [13].
**Monitoring of antimicrobial resistance**	We propose that resistance levels in the community could be monitored using sentinel general practices to systematically sample infections or even uninfected attending patients. Routine monitoring resistance in aerobes (collected by nasal swabs) should be straightforward— although anaerobes (collected by faecal swabs) would be more difficult [14].<br>Particularly important gaps in knowledge that need to be filled include the following: Information on: the incidence, prevalence, range across pathogens and geographical patterns related to antimicrobial resistance is needed to be made accessible in a timely manner in order to guide the treatment of patients; to inform local, national and regional actions; and to monitor the effectiveness of interventions…[3].<br><br>**Secondary question: Which stakeholders have responsibility for developing and implementing monitoring of general practice antibiotic resistance?**<br>The [clinical microbiology service] should provide annual analyses of cumulative AMR to groups with responsibility for local antimicrobial therapy guidelines to inform recommendations for local empirical therapy and formulary management [4].<br>Develop a national surveillance system for antimicrobial resistance that: includes a national reference centre with the ability systematically to collect and analyse data – including those on a core set of organisms and antimicrobial medicines from both health care facilities and the community – in order to inform national policies and decision-making; includes at least one reference laboratory capable of susceptibility testing to fulfil the core data requirements, using standardized tests for identification of resistant microorganisms and operating to agreed quality standards [3].

**Feedback to prescribers and reporting**	The how and why of measurement in antimicrobial stewardship is important but more important is that once you have gone to the effort to collect and analyse the data that you use it, that you share it with front-line clinicians to enable them to reflect on their practice and change their prescribing behaviour to improve patient outcomes and minimise resistance and other harm. It is important to share data in as near real time as possible…Comparison with peers and identification of prescribers who are outliers are useful techniques to change behaviour… [9].<br>Consider developing systems and processes for providing regular updates (at least every year) to individual prescribers and prescribing leads on: local and national antimicrobial resistance rates and trends; individual prescribing benchmarked against local and national antimicrobial prescribing rates and trends; patient safety incidents related to antimicrobial use, including hospital admissions for potentially avoidable life-threatening infections, infections with C. difficile or adverse drug reactions such as anaphylaxis [6].
**2.1.3. Education**	
**Community & patient education about AMR and AMS**	Consumers should be provided with information about the risks and benefits of the most effective and appropriate treatment options for them. This includes information about specific antimicrobials (if appropriate) and the risks associated with AMR. When discussing the use of antimicrobials and AMR with consumers, it is important that the messages are clear, simple and consistent. Information may need to be provided in different formats and styles, tailored to the needs and preferences of the consumer [4].<br>Objective 1: Improve awareness and understanding of antimicrobial resistance through effective communication, education and training. Steps need to be taken immediately in order to raise awareness of antimicrobial resistance and promote behavioural change, through public communication programmes that target different audiences in human health, animal health and agricultural practice as well as consumers. Inclusion of the use of antimicrobial agents and resistance in school curricula will promote better understanding and awareness from an early age [3].<br><br>**Secondary question: Which stakeholders have responsibility for implementing community and patient education about AMR and AMS?**<br>All staff members in outpatient facilities, including administrative staff members, medical assistants, nurses, allied health professionals, and medical directors, can improve antibiotic prescribing by using consistent messages when communicating with patients about the indications for antibiotics [11].<br>Other stakeholders – including civil society organizations, trade and industry bodies, employee organizations, foundations with an interest in science education, and the media – should help to promote public awareness and understanding of infection prevention and use of antimicrobial medicines across all sectors [3].
**GP continuing education in AMS & AMR**	For clinicians, AMS education should start during undergraduate training and continue throughout their careers. Local education programs should include local AMS recommendations. Programs that are multifaceted and include one or more active educational activities are more likely to be successful in changing clinicians' behaviour [4].<br>Support multiprofessional local groups in the implementation of infection treatment recommendations, e.g. by producing locally adapted materials and local educational meetings and events [10].<br><br>**Secondary question: Which stakeholders have responsibility for implementing GP continuing education?**<br>Professional colleges and associations can take a proactive role in supporting AMS – for example, by updating their members about changes to guidelines and providing continuing education or discussion forums. … A multidisciplinary group that includes ID physicians, clinical microbiologists, clinical pharmacists, nurses, midwives and infection control practitioners, or the AMS team, should be responsible for planning, developing and delivering a local education program. This will help to ensure that the approach to education is suitable for the intended audience and relevant to the local practice context [4].
**GP education on communication skills, patient-centred approaches & shared decision making**	Communications skills training can be used to promote strategies to address patient concerns regarding prognosis, benefits, and harms of antibiotic treatment; management of self-limiting conditions; and clinician concerns regarding managing patient expectations for antibiotics during a clinical visit [11].

**GP education about non-antibiotic management of self-limiting infection**	If antimicrobial treatment is not considered necessary, give the patient advice about the expected natural history of the illness, the limited or absent benefit of antimicrobial treatment, and the potential unwanted side effects of antimicrobials such as diarrhoea and rash, recommendations for symptom management, as well as advice about actions in case of worsening clinical condition (safety netting) [8].<br><br>If immediate antimicrobial prescribing is not the most appropriate option, discuss with the patient and/or their family members or carers (as appropriate) other options such as self-care with over-the-counter preparations, back-up (delayed) prescribing, other non-pharmacological interventions, for example, draining the site of infection [6].
**GP education about delayed prescribing or watchful waiting**	Senior leaders, including medical directors, Clinical Commissioning Group (CCG) chairs and directors of public health, need to support and empower prescribers, and other health and public health professionals who advise on prescribing decisions, to make the decision not to prescribe where other appropriate strategies exist such as 'watchful waiting' or delayed prescribing [7].
**General practice team member education**	Communicate with all clinic staff members to set patient expectations. Patient visits for acute illnesses might or might not result in an antibiotic prescription. All staff members in outpatient facilities, including administrative staff members, medical assistants, nurses, allied health professionals, and medical directors, can improve antibiotic prescribing by using consistent messages when communicating with patients about the indications for antibiotics. Education for clinicians and clinic staff members can reinforce appropriate antibiotic prescribing and improve the quality of care [11].
**Independent education (restrict pharma marketing)**	Drug advertising and academic detailing by pharmaceutical companies influences physicians prescribing behaviours. Further regulation of the material supplied to clinicians by the pharmaceutical industry may be needed if overuse of broad-spectrum antimicrobials is to be reduced. [15].<br>In some cases, industry spending on promoting products is greater than governmental investment in promoting rational use of antimicrobial medicines or providing objective information…Professional bodies and associations, including industry associations, health insurance providers and other payers, should develop a code of conduct for appropriate training in, education about, and marketing, purchasing, reimbursement and use of antimicrobial agents. This code should include commitment to comply with national and international regulations and standards, and to eliminate dependence on the pharmaceutical industry for information and education on medicines and, in some cases, income [3].
**2.1.4. Consultation support**	
**Prescribing guidelines**	Evidence-based prescribing guidelines for antimicrobials are a fundamental component of AMS programs because they guide appropriate antimicrobial use. They can also be used to educate prescribers and students on accepted practice for antimicrobial prescribing in the organisation…This includes the importance of documenting in the patient's healthcare record the indication for the prescribing decision and, where the prescriber varies from guideline-concordant practice, the rationale for the decision [4].<br>Organizational structures could pose barriers to [AMS]. Clinic visits were often too brief to discuss guidelines with patients…External guidelines were not always used because they were sometimes difficult to locate, too long, or not seen as relevant…external guidelines needed to be accessible to clinicians and trusted by clinicians [16].<br>…national treatment guidelines must include: diagnostic criteria for each condition; an analysis of the antibiotic risks and benefits both for the patient and for society; and recommendations for when to reevaluate a patient's treatment. Second, to ease the implementation of national guidelines in primary health care, they need to be transformed into simple treatment algorithms, e.g. clear advice to health professionals on when and when not to prescribe an antibiotic…easily accessible summaries of guidelines for common infections have been well received and used [10].<br><br>**Secondary question: Which stakeholders have responsibility for implementing prescribing guidelines?**<br>In 2010 the UK Health Protection Agency developed and updated antibiotic guidance for GPs, which was locally adaptable by primary care trusts and distributed to practices [1].<br>Require explicit written justification in the medical record for nonrecommended antibiotic prescribing. This technique has reduced inappropriate prescribing by holding clinicians accountable in the medical record for their decisions [11].

**Point of care tests**	In POC testing interventions, participants found that it was unclear which staff members to train in POC testing, as various organizational roles performed the test in different clinics [16].<br>A disadvantage of near patient testing is that it may increase patients' expectations and increase re-consultation by medicalising self limiting illnesses such as sore throat [15].<br>Decisions to prescribe antibiotics are rarely based on definitive diagnoses. Effective, rapid, low-cost diagnostic tools are needed for guiding optimal use of antibiotics in human and animal medicine, and such tools should be easily integrated into clinical, pharmacy and veterinary practices [3].
**Microbiology testing and reporting**	Microbiology testing is a key component of antimicrobial stewardship (AMS). The clinical microbiology service (CMS) performs the combined role of patient-specific diagnostic testing to guide direct patient care, and system-wide diagnostic stewardship, surveillance of resistant organisms and outbreak investigation. a positive microbiology diagnostic test is used to confirm a provisional clinical diagnosis, and the antimicrobial susceptibility results guide targeted antimicrobial management. Optimal specimen collection and transport are critical elements of the testing process. [4].<br>Restrictive reporting of the results of antimicrobial susceptibility testing is one stewardship activity that varies from laboratory to laboratory and, perhaps, may be underused… [9].<br>Considering that most bacterial infections are also self-limiting (5), antibiotic prescription on the basis of a positive result in an otherwise healthy individual should be carefully considered [2].
**Allergy testing**	Promote allergy testing for patients with a history of allergic reaction to beta-lactams, as a measure to promote use of first-line antimicrobials in non-allergic patients [8].
**Electronic decision support for prescribers**	eCDSSs [Electronic clinical decision support systems] can organise and present appropriate information to the user in a way that supports them to make clinical decisions with increased accuracy and reduced error… may include online access to documents such as formulary restrictions, local antimicrobial prescribing guidelines and Therapeutic Guidelines: Antibiotic [4].<br>Advanced decision support systems use complex logic, mathematical modelling or case-based probabilities to provide patient-specific recommendations. They can provide decision support by helping identify potential infections, pathogens and treatment options based on inputs about patient symptoms… CDSSs are simply assistive tools and cannot replace expert decision-making. They may support the prescriber or the AMS program, or both [9].
**Expert advice**	Clinicians may also want to discuss antimicrobial prescriptions with nominated experts based on clinical concerns. Pathways for prescribers in community settings to access such specialist advice should be clearly identified. This may occur through links with ID or pharmacy services at local hospitals, or with clinical microbiologists at laboratory service providers…Telehealth can support improved access to clinical services, specialist advice, diagnostic information and education, over distance, as part of formalised service networks [4].<br>Strategies to encourage appropriate prescribing in primary care include the development of evidence-based policies, in collaboration with local experts, who provide practical guidance on how to rule out serious infections and how to handle patient demand for an antibiotic, complemented by information on various symptomatic treatment options [2].<br>Clinical microbiologists should be available to clinicians for counselling on diagnostics of infectious diseases, including correct sampling and interpretation of test results, difficult-to-treat pathogens and complicated infections. Pharmacists in community and hospital settings have expertise in medicines and are the gatekeepers to the use of antimicrobials. As such, pharmacists can act as an important source of advice and information for patients and prescribers on the safe, rational and effective use of antimicrobials [8].<br>Telephone advice lines are provided in a few regions of France. These are provided by hospitals or health networks and may be staffed by an infectious disease specialist or a trained GP [13].
**Decision support for use with patients**	Providing easy-to-understand information to consumers about the expected duration of symptoms, and how to identify signs and symptoms of more serious illness, may help to manage their expectations about antimicrobials. Consumers should be provided with information about the risks and benefits of the most effective and appropriate treatment options for them... When discussing the use of antimicrobials and AMR with consumers, it is important that the messages are clear, simple and consistent. Information may need to be provided in different formats and styles, tailored to the needs and preferences of the consumer [4].<br>Patient information leaflets on common infections are produced in six languages to target a large proportion of the immigrant population [10].
**2.1.5. Pharmacy and nursing approaches**	
**Unit dispensing**	Explore per unit dispensing of antimicrobials taking into consideration all relevant guidelines and regulations [8].

**Supply of and timely access to antibiotics**	Ensure the adequate supply of, and timely access to, antimicrobials…Certain interventions, such as removing broad-spectrum antimicrobials from clinical areas to limit their inappropriate use, may delay antimicrobial delivery if appropriate pathways for antimicrobial supply do not accompany the restrictions [4].<br>Ensure access to the antimicrobials recommended in clinical guidance, by conducting a review of national market availability, implementing measures to support sustained market availability for both innovative and generic products and tackling shortages. At the same time, limit the use of last-resort antimicrobials to safeguard their effectiveness, by establishing restrictive measures for use… [8].
**Pharmacy review & advice**	In addition to clinically reviewing and dispensing antimicrobial prescriptions, community pharmacists can educate patients and carers about using antimicrobials appropriately…Pharmacists should consider whether there is still a clinical need to fill all prescriptions presented – for example, original and repeat prescriptions that are presented for dispensing several months after they were written (when it would be expected that the original infection would have resolved), or prescriptions for long-term use (for example, for several months). Such prescriptions should only be dispensed if the pharmacist is satisfied that the use is appropriate. If not, there should be discussion with the prescriber. Community pharmacy is an important site of community education and activities for AMS in primary care because of the ease and frequency of the public's access to community pharmacists compared with other clinicians…At the system level, the pharmacist's role may include planning and implementing AMS programs and other initiatives that encourage appropriate antimicrobial use [4].<br>It is important that any advice on medications and formulations is tailored to the patient's specific symptoms and preferences. In addition, pharmacy staff need to be able to identify red-flag symptoms and other risk factors for a serious infection and refer patients to physicians where necessary… [2].
**Appropriate disposal of left-over antibiotics**	It should be routine practice that consumers who have been dispensed antimicrobials, or their carers, are…Advised not to keep any unused antimicrobials, but to return them to a pharmacy for disposal [4].
**Nurse triage, patient assessment & education**	…professional associations and experts, internationally and in Australia, highlight that nurses, midwives and infection control practitioners (ICPs) play key roles in preventing and controlling AMR. They can help to safeguard the effectiveness of antimicrobials through infection prevention and control, education, and involvement in AMS activities. [4].<br>Materials are created for nurses providing education about common infections to parents of newborns at child health-centres and for schoolchildren… [10].<br>Use call centers, nurse hotlines, or pharmacist consultations as triage systems to prevent unnecessary visits [11].
**2.1.6. Research**	
**Research into AMR/AMS gaps, translation into practice**	Agree a national research agenda and promote investment in innovative approaches to containing antimicrobial resistance…Priority areas for action are to: Identify current gaps, and agree to national research and development priorities… More research is needed to understand any unintended consequences of the use of restrictive interventions [4].<br>Few studies focused on the organization component of the work system model or the structures and roles that organize a clinic… [16].<br>There is a paucity of studies on the potential harm of withholding or overuse of antibiotics and how to identify which patients may benefit, and by how much [15].<br>…implementation research is needed to determine which outpatient stewardship interventions work best in different outpatient settings, effective strategies to implement interventions, and sustainable approaches to outpatient stewardship [11].
**Research into context, culture of general practice and behaviour change strategies**	In general, prescribing has been shown to be influenced by several factors, including the cultural beliefs of the patient and the prescriber, patient demand, socio-economic factors and clinical autonomy. [12].<br>Understanding the organisational context, culture and workplace norms, including local prescribing rules and behaviours, is critical to successfully establishing an AMS program. A 'one size fits all' approach is not appropriate and does not sufficiently recognise that each setting has unique elements to be considered, such as enablers and barriers for appropriate antimicrobial prescribing and use… Education strategies that incorporate behaviour change principles such as audit and feedback, along with more active strategies including academic detailing, consensus-building sessions and educational workshops, are more effective in changing behaviour than the passive dissemination of information alone [4].

References

1. Ashiru-Oredope, D.; Sharland, M.; Charani, E.; McNulty, C.; Cooke, J.; ARHAI Antimicrobial Stewardship Group. Improving the quality of antibiotic prescribing in the NHS by developing a new Antimicrobial Stewardship Programme: Start Smart - Then Focus. *J. Antimicrob. Chemother.* **2012**, *67 Suppl 1*, i51-63, doi:10.1093/jac/dks202.
2. Essack, S.; Pignatari, A.C. A framework for the non-antibiotic management of upper respiratory tract infections: towards a global change in antibiotic resistance. *Int. J. Clin. Pract. Suppl.* **2013**, *67* 4-9, doi:10.1111/ijcp.12335.
3. World Health Organization. Global action plan on antimicrobial resistance. WHO: Geneva, 2015.
4. Australian Commission on Safety and Quality in Health Care. Antimicrobial stewardship in Australian health care. ACSQHC: Sydney, 2018.
5. Molstad, S.; Erntell, M.; Hanberger, H.; Melander, E.; Norman, C.; Skoog, G.; Lundborg, C.S.; Söderström, A.; Torell, E.; Cars, O. Sustained reduction of antibiotic use and low bacterial resistance: 10-year follow-up of the Swedish Strama programme. *Lancet Infect. Dis.* **2008**, *8*, 125-132, doi:10.1016/s1473-3099(08)70017-3.
6. National Institute for Health and Care Excellence. Antimicrobial stewardship: systems and processes for effective antimicrobial medicine use. Full guideline: methods, evidence and recommendations. In *NICE guideline*, NICE: 2015.
7. The UK Faculty of Public Health; The Royal College of Physicians; The Royal Pharmaceutical Society; The Royal College of Nursing; The Royal College of General Practitioners. Joint statement on antimicrobial resistance. FPH, RCP, RPS, RCN, RCGP: 2014.
8. European Commission. EU Guidelines for the prudent use of antimicrobials in human health. ECDC: 2017.
9. British Society for Antimicrobial Chemotherapy; ESCMID Study Group for Antimicrobial Stewardship; European Society of Clinical Microbiology and Infectious Diseases. Antimicrobial stewardship: from principles to practice. BSAC: Birmingham, 2018.
10. Molstad, S.; Lofmark, S.; Carlin, K.; Erntell, M.; Aspevall, O.; Blad, L.; Hanberger, H.; Hedin, K.; Hellman, J.; Norman, C., et al. Lessons learnt during 20 years of the Swedish strategic programme against antibiotic resistance. *Bull. World Health Organ.* **2017**, *95*, 764-773, doi:10.2471/BLT.16.184374.
11. Sanchez, G.V.; Fleming-Dutra, K.E.; Roberts, R.M.; Hicks, L.A. Core elements of outpatient antibiotic stewardship. *MMWR Recomm. Rep.* **2016**, *65*, 1-12, doi:10.15585/mmwr.rr6506a1.
12. Ashiru-Oredope, D.; Hopkins, S.; English Surveillance Programme for Antimicrobial Utilization Resistance Oversight Group. Antimicrobial stewardship: English Surveillance Programme for Antimicrobial Utilization and Resistance (ESPAUR). *J. Antimicrob. Chemother.* **2013**, *68*, 2421-2423, doi:10.1093/jac/dkt363.
13. Wang, S.; Pulcini, C.; Rabaud, C.; Boivin, J.M.; Birge, J. Inventory of antibiotic stewardship programs in general practice in France and abroad. *Med. Mal. Infect.* **2015**, *45*, 111-123, doi:10.1016/j.medmal.2015.01.011.
14. Del Mar, C.B.; Scott, A.M.; Glasziou, P.P.; Hoffmann, T.; van Driel, M.L.; Beller, E.; Phillips, S.M.; Dartnell, J. Reducing antibiotic prescribing in Australian general practice: time for a national strategy. *Med. J. Aust.* **2017**, *207*, 401-406, doi:10.5694/mja17.00574.
15. McNulty, C.A. Optimising antibiotic prescribing in primary care. *Int. J. Antimicrob. Agents* **2001**, *18*, 329-333, doi:10.1016/s0924-8579(01)00412-5.
16. Keller, S.C.; Tamma, P.D.; Cosgrove, S.E.; Miller, M.A.; Sateia, H.; Szymczak, J.; Gurses, A.P.; Linder, J.A. Ambulatory antibiotic stewardship through a human factors engineering approach: a systematic review. *J. Am. Board Fam. Med.* **2018**, *31*, 417-430, doi:10.3122/jabfm.2018.03.170225.

Supplementary file 3 Search strategy

The websites searched

- Australian Commission on Safety and Quality in Health Care (ACSQHC) www.safetyandquality.gov.au
- British Society for Antimicrobial Chemotherapy www.bsac.org.uk
- Centres for Disease Control and prevention www.cdc.gov
- European Centre for Disease Prevention and Control https://ecdc.europa.eu/en/home
- National Health Service www.nhs.uk
- National Institute for Health and Care Excellence www.nice.org.uk
- Royal Australian College of General Practitioners www.racgp.org.au
- Royal College of General Practitioners www.rcgp.org.uk
- World Health Organization www.who.int

Embase via Ovid; 1974 to 21 Sept 2018

1. Index term: exp antibiotic agent/
2. Text terms: (Antibacterial? OR Anti-bacterial? OR Antibiotic? OR Anti-biotic? OR Antimicrobial? OR Anti-microbial?).mp
3. **1 OR 2 [Antibiotics**]
4. Index: exp Prescription/
5. Text: (prescrib* OR prescrip* OR antibiotic therapy OR antibiotic treatment OR antibiotic prescription$).mp
6. **4 OR 5 [Antibiotic prescriptions]**
7. Index term: exp general practitioner/
8. Text terms (general practitioner$ OR family medicine practitioner$ OR family medicine physician$ OR family physician$).mp
9. **7 OR 8 [GPs]**
10. Index term: exp primary medical care/ OR exp primary health care/ OR exp ambulatory care/ OR exp outpatient care/ OR exp general practice/
11. Text terms: (primary care OR primary health care OR primary healthcare OR outpatient? OR office visit* OR ambulatory care facilities OR community health cent* OR ambulatory care OR general practice OR family practice).mp
12. **10 OR 11 [General practice]**
13. Index: exp inappropriate prescribing/ OR exp practice guideline/
14. Text: (stewardship OR inappropriate prescribing OR antibiotic overuse OR formulary restriction OR restrictive strateg* OR restrictive polic* OR optimi#ation OR authori#ation OR guideline).mp
15. **13 OR 14 [Stewardship**]
16. **3 AND 6 AND 9 AND 12 AND 15 [Antibiotic prescribing by GPs in general practice with AMS]**
17. Index term: Exp Health care policy/ OR exp accreditation /OR exp Health care quality/
18. Text: (framework OR approach OR model OR system OR policy OR strategy).mp
19. **17 OR 18 [Policy + Framework]**
20. **16 AND 19 [GPs+ AMS + gen practice +framework]**

Ovid MEDLINE(R) 1946 to 21 Sept 2018

1. exp Anti-Bacterial Agents/ OR (Antibacterial? OR Anti-bacterial? OR Antibiotic? OR Anti-biotic? OR Antimicrobial? OR Anti-microbial?).mp
2. exp Prescriptions/ OR (prescrib* OR prescrip* OR antibiotic therapy OR antibiotic treatment OR antibiotic prescription$).mp
3. exp general practitioners/ OR exp physicians, family/ OR exp physicians, primary care/ OR (general practitioner$ OR family medicine practitioner$ OR family medicine physician$ OR family physician$ OR physician$).mp
4. exp general practice/ OR exp family practice/ OR (primary care OR primary health care OR primary healthcare OR outpatients OR office visit* OR ambulatory care facilities OR community health cent* OR ambulatory care OR general practice OR family practice).mp
5. exp Antimicrobial Stewardship/ OR exp inappropriate prescribing/ OR exp Guideline Adherence/ OR (stewardship OR inappropriate prescribing OR antibiotic overuse OR formulary restriction OR restrictive strateg* OR restrictive polic* OR optimi#ation OR authori#ation OR guideline OR program* OR standard$).mp
6. exp Health Policy/ OR exp accreditation/ OR exp Quality of Health Care/ OR (framework OR approach OR model OR systems OR policy OR strategy).mp

1 AND 2 AND 3 AND 4 AND 5 AND 6

PsycINFO; 1806 to Sept week 3, 2018

exp Antibiotics/
(Antibacterial? OR Anti-bacterial? OR Antibiotic? OR Anti-biotic? OR Antimicrobial? OR Anti-microbial?).mp
1 OR 2 [Antibiotics]
exp "PRESCRIBING (DRUGS)"/
(prescrib* OR prescrip* OR antibiotic therapy OR antibiotic treatment OR antibiotic prescription$).mp
4 OR 5 [Antibiotic prescriptions/prescribing]
exp general practitioners/ OR exp family physicians/
(general practitioner$ OR family medicine practitioner$ OR family medicine physician$ OR family physician$).mp
7 OR 8 [GPs]
exp primary health care/ OR exp outpatients / OR exp outpatient treatment / OR family medicine/
(primary care OR primary health care OR primary healthcare OR outpatient? OR office visit* OR ambulatory care facilities OR community health cent* OR ambulatory care OR general practice OR family practice).mp
10 OR 11 [General practice]
exp treatment guidelines/ OR exp Evidence based practice/
(stewardship OR inappropriate prescribing OR antibiotic overuse OR formulary restriction OR restrictive strateg* OR restrictive polic* OR optimi#ation OR authori#ation OR guideline? OR evidence based practice OR program*).mp
13 OR 14 [Stewardship]
3 AND 6 AND 9 AND 12 AND 15 [Antibiotic prescribing by GPs in general practice with AMS] (18 results only)
exp quality control/ or exp "quality of care"/ or exp "quality of services"/
(framework OR approach OR model OR system OR policy OR strategy)
17 OR 18
16 AND 19

EBSCOhost CINAHL Plus; 1997 - 20 May 2018
(MH "Antibiotics+") OR (Antibacterial* OR Anti-bacterial* OR Antibiotic* OR Anti-biotic* OR Antimicrobial* OR Anti-microbial*)
AND
(MH "Prescriptions, Drug") OR (MH "Drugs, Prescription") OR (prescrib* OR prescrip* OR "antibiotic therapy" OR "antibiotic treatment" OR "antibiotic prescription*")
AND
(MH "Physicians, Family") OR ("general practitioner*" OR "family medic* practitioner*" OR "family medic* physician*" OR "family physician*")
AND
(MH "Primary Health Care") OR (MH "Outpatients") OR (MH "Ambulatory Care Facilities") OR (MH "Outpatient Service") OR (MH "Family Practice") OR (MH "Ambulatory Care") OR (MH "Community Health Centers+") OR ("primary care" OR "primary health care" OR "primary healthcare" OR outpatient* OR "office visit*" OR "ambulatory care facilities" OR "community health cent*" OR "ambulatory care" OR "general practice" OR "family practice")
AND
(MH "Inappropriate Prescribing") OR (MH "Guideline Adherence") OR (MH "Prescribing Patterns") OR (stewardship OR "inappropriate prescribing" OR "antibiotic overuse" OR "formulary restriction" OR "restrictive strateg*" OR "restrictive polic*" OR optimi#ation OR authori#ation OR guideline OR program*)
AND
(MH "Quality of Health Care+") OR (MH "Accreditation") OR (framework OR approach OR model OR system* OR policy OR strategy)

Scopus; Searched on 11/10/18.
Antibacterial* OR Anti-bacterial* OR Antibiotic* OR Anti-biotic* OR Antimicrobial* OR Anti-microbial*
AND
prescrib* OR prescrip* OR "antibiotic therapy" OR "antibiotic treatment" OR "antibiotic prescription*"
AND
"general practitioner*" OR "family medicine practitioner*" OR "family medicine physician*" OR "family physician*"
AND
"primary care" OR "primary health care" OR "primary healthcare" OR outpatients OR "office visit*" OR "ambulatory care facilit*" OR "community health cent*" OR "ambulatory care" OR "general practice*" OR "family practice*"
AND
stewardship OR "inappropriate prescribing" OR "antibiotic overuse" OR "formulary restriction" OR "restrictive strateg*" OR "restrictive polic*" OR optimi#ation OR authori#ation OR guideline OR program* OR standard$
AND
Framework* OR approach OR model OR system* OR policy OR strategy

Cochrane Database

1. MeSH descriptor: [Anti-Bacterial Agents] explode all trees
2. (Antibacterial? OR Anti-bacterial? OR Antibiotic? OR Anti-biotic? OR Antimicrobial? OR Anti-microbial?):ti,ab,kw
3. #1 or #2
4. MeSH descriptor: [Prescriptions] explode all trees
5. (prescrib* OR prescrip* OR antibiotic therapy OR antibiotic treatment OR antibiotic prescription$):ti,ab,kw
6. #4 OR #5
7. MeSH descriptor: [General Practitioners] explode all trees
8. MeSH descriptor: [Physicians, Family] explode all trees
9. MeSH descriptor: [Physicians, Primary Care] explode all trees
10. (general practitioner$ OR family medicine practitioner$ OR family medicine physician$ OR family physician$):ti,ab,kw
11. #7 OR #8 OR #9 OR 10
12. MeSH descriptor: [General Practice] explode all trees
13. MeSH descriptor: [Primary Health Care] explode all trees
14. MeSH descriptor: [Family Practice] explode all trees
15. (primary care OR primary health care OR primary healthcare OR outpatient? OR office visit* OR ambulatory care facilities OR community health cent* OR ambulatory care OR general practice OR family practice):ti,ab,kw
16. #12 OR #13 OR #14 OR #15
17. MeSH descriptor: [Antimicrobial Stewardship] explode all trees
18. MeSH descriptor: [Inappropriate Prescribing] explode all trees
19. (stewardship OR inappropriate prescribing OR antibiotic overuse OR formulary restriction OR restrictive strateg* OR restrictive polic* OR optimization OR authorization OR guideline OR optimisation OR authorisation):ti,ab,kw
20. #17 OR #18 OR #19
21. #3 AND #6 AND #11 AND #16 AND #20
22. MeSH descriptor: [Health Policy] explode all trees
23. MeSH descriptor: [Quality of Health Care] explode all trees
24. (framework OR approach OR model OR system OR policy OR strategy). ti,ab,kw
25. #22 OR #23 OR #24
26. #21 AND #25
